# Randomized Phase 2 Trial of an Extended and Flexible Dosing Schedule of ^177^Lu-PSMA Molecular Radiotherapy in Patients with Metastatic Castration-Resistant Prostate Cancer (FLEX-MRT): Study Protocol

**DOI:** 10.2967/jnumed.125.269495

**Published:** 2025-10

**Authors:** Adrien Holzgreve, Astrid Delker, Zachary Ells, Julia Brosch-Lenz, Lena M. Unterrainer, John Nikitas, Shaojun Zhu, Maria M. Contreras, Hamzah Alam, Rejah M. Nabong, Stephanie Lira, Arseniy Vasilyev, Lillian Chen, Tristan Grogan, David Elashoff, Catherine A. Meyer, Magnus Dahlbom, Johannes Czernin, Jérémie Calais

**Affiliations:** 1 Ahmanson Translational Theranostics Division, Department of Molecular and Medical Pharmacology, David Geffen School of Medicine, UCLA, Los Angeles, California;; 2 Department of Nuclear Medicine, LMU University Hospital, LMU Munich, Munich, Germany;; 3 Institute of Nuclear Medicine Inc., Bethesda, Maryland;; 4 Bavarian Cancer Research Center, Partner Site Munich, Munich, Germany; and; 5 Department of Medicine Statistics Core, Department of Medicine’s Division of General Internal Medicine and Health Services Research, David Geffen School of Medicine, UCLA, Los Angeles, California

**Keywords:** metastatic castration-resistant prostate cancer, mCRPC, [^177^Lu]Lu-PSMA-617, radiopharmaceutical therapy, RPT, treatment holidays, prostate-specific membrane antigen, PSMA, SPECT/CT, therapy response assessment

## Abstract

[^177^Lu]Lu-PSMA-617 radiopharmaceutical therapy has been approved for the treatment of men with metastatic castration-resistant prostate cancer (mCRPC) using a fixed dosing schedule of once every 6 wk for up to a total of 6 doses. We hypothesized that patients may benefit from a flexible and extended dosing schedule, up to 12 doses with potential “treatment holiday” periods. **Objective:** The objective of this study is to determine the 2-y survival rate of patients with mCRPC treated with an extended and flexible dosing schedule of [^177^Lu]Lu-PSMA-617 therapy in comparison to patients treated with the standard fixed dosing schedule of a maximum of 6 treatment cycles once every 6 wk. **Study Design:** The FLEX-MRT trial is an investigator-initiated prospective phase 2, parallel group, randomized, controlled, open-label, single-center trial in men with mCRPC to determine the efficacy of a flexible and extended dosing schedule of [^177^Lu]Lu-PSMA-617 therapy. Key inclusion criteria are patients eligible for Pluvicto (i.e., prior androgen receptor signaling inhibitors, prior chemotherapy, PSMA PET VISION criteria). Key exclusion criteria are prior [^177^Lu]Lu-PSMA-617 therapy and less than 6 wk since last myelosuppressive therapy. The trial aims to centrally randomize 90 patients in a 1:1 ratio to 2 treatment arms. In the control arm, patients will be treated with the approved standard dosing schedule (*n* = 45). In the investigational arm, patients will be treated with up to 12 cycles and with potential treatment holidays depending on response (*n* = 45). Response assessment is based on SPECT/CT at each cycle and on PSMA PET/CT during treatment holiday periods (every 12 wk). Primary endpoint is the 2-y survival rate. Survival is calculated from the date of the first cycle of [^177^Lu]Lu-PSMA-617 therapy. Secondary endpoints include safety by Common Terminology Criteria for Adverse Events and dosimetry and determination of overall and progression-free survival (evidence of progression as defined by radiographic, prostate-specific antigen level, or clinical progression, or death from any cause).

Radiopharmaceutical therapy (RPT) with [^177^Lu]Lu-PSMA-617 was approved both by the U.S. Food and Drug Administration and the European Medicines Agency in 2022 for the treatment of patients with metastatic castration-resistant prostate cancer (mCRPC) following the VISION trial (1,2). The VISION trial demonstrated that ^177^Lu-PSMA RPT can improve overall survival, radiographic progression-free survival (PFS), prostate-specific antigen (PSA) response, quality of life, and bone pain in patients with progressing mCRPC in comparison to the standard of care (2,3). However, even after a good response to [^177^Lu]Lu-PSMA-617 therapy, patients will eventually relapse and further treatment opportunities are limited. Hence, further optimization of the treatment is warranted.

Following the VISION trial design, [^177^Lu]Lu-PSMA-617 was approved by the Food and Drug Administration for patients with mCRPC on a fixed dosing schedule: up to 6 doses (cycles) of [^177^Lu]Lu-PSMA-617 at an injected activity of 7.4 GBq, administered at a time interval of 6 wk.

Retreatment (after an interval without ^177^Lu-PSMA, with or without other therapy) and extended regimen (>6 cycles) of ^177^Lu-PSMA therapy have been performed successfully outside of the United States with favorable efficacy and toxicity profiles (4–9). There are no guidelines for the optimum number of cycles of ^177^Lu-PSMA in patients who show good response. Because of the approved drug label and insurance coverage, ^177^Lu-PSMA is usually discontinued after a maximum number of 6 cycles. Moreover, some patients experience a complete or almost complete response (CR) before the last cycle 6 (e.g., after cycle 2). It is unclear whether these patients benefit from the subsequent remaining treatment cycles. In these patients with no visible lesions anymore on PET or SPECT or with limited or minimal residual lesions, the risk–benefit ratio may not be in favor of administering the subsequent treatment cycles. ^177^Lu-PSMA therapy may be saved for later time points, when the disease becomes again visible and large enough to be treated with β-radiation. A treatment holiday could reduce patients’ exposure to the therapy and prevent unnecessary toxicity when treatment seems to be already maximally effective, with potentially no further benefit expected from further treatment cycles. The concept of treatment holidays in patients experiencing a complete or almost CR before the last cycle 6 (e.g., after cycle 2) has been reported by Emmett et al. and Seifert et al. (7,10).

In the randomized phase 2 trial FLEX-MRT (ClinicalTrials.gov identifier: NCT06216249), we aimed at assessing prospectively the concept of a flexible and extended (>6 cycles) regimen of ^177^Lu-PSMA RPT that includes potential treatment holiday periods, and up to 12 cycles.

## MATERIALS AND METHODS

The full study protocol and the SPIRIT guidelines checklist are provided as supplemental materials (available at http://jnm.snmjournals.org) (11).

## STUDY DESIGN

### Overall Study Design

This is a prospective, open-label, randomized, controlled, parallel group, single-center phase 2 trial of a flexible and extended dosing schedule of [^177^Lu]Lu-PSMA-617 therapy in comparison to the standard fixed dosing schedule in patients with mCRPC.

The study was powered to demonstrate a survival benefit of the flexible and extended dosing schedule in comparison to the standard fixed dosing schedule. We hypothesized a 2-y survival rate of 55% in the investigational group and 30% in the control group (2). The overall sample size is 90 patients (45 in the control group and 45 in the treatment group) to achieve 80% power. The planned study duration is 48 mo.

The flexible dosing schedule in the investigational arm will be based on SPECT/CT response assessments obtained 24 h after the injection of [^177^Lu]Lu-PSMA-617. The response assessment during treatment holiday periods will be based on prostate-specific membrane antigen (PSMA) PET/CT every 12 wk using RECIP guidelines.

Single-time-point SPECT/CT dosimetry protocol at every cycle will be performed and will allow the number of cycles that subjects may receive under the study without exceeding the kidney dose threshold to be determined. An interim safety analysis with a stopping rule based on safety outcomes is planned at 50% of enrollment.

**FIGURE 1.**
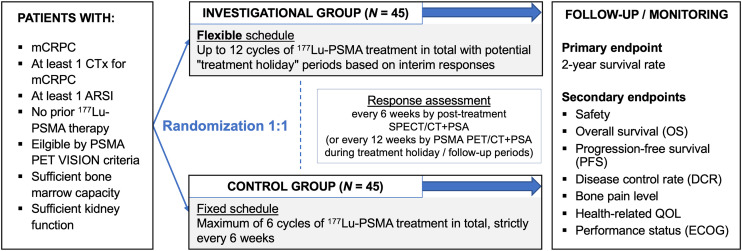
Study design. FLEX-MRT trial will randomize 90 patients in 1:1 ratio to investigational arm treated with extended and flexible dosing schedule and control arm treated with approved standard dosing schedule. Primary endpoint is 2-y survival rate. ARSI = androgen receptor signaling inhibitor; CTx = chemotherapy; QOL = quality of life.

The overall study design is shown in Figure 1.

### Trial Registration

The FLEX-MRT trial is taking place at the Ahmanson Translational Theranostics Division at the University of California Los Angeles (UCLA). FLEX-MRT is an investigator-initiated trial supported by Novartis, conducted under the Investigational New Drug Application 168362 and approved by the UCLA Institutional Review Board 23-000931. The trial is registered at ClinicalTrials.gov (National Clinical Trial identifier NCT06216249, first posted January 22, 2024).

## STUDY ENDPOINTS

### Primary Endpoint

The primary endpoint is the 2-y survival rate of patients treated with [^177^Lu]Lu-PSMA-617 on a flexible and extended dosing schedule (investigational arm) in comparison to patients treated with the standard fixed dosing schedule of maximum 6 treatment cycles every 6 wk (control arm). Survival is calculated from the date of the first cycle of [^177^Lu]Lu-PSMA-617 therapy.

### Secondary Endpoints

The secondary endpoints included the following.

1. To evaluate the safety of a flexible dosing schedule of [^177^Lu]Lu-PSMA-617 including up to 12 cycles and “treatment holiday” periods by Common Terminology Criteria for Adverse Events guidelines and dosimetry.
2. To determine the overall survival, defined as the time from the date of the first cycle injection of [^177^Lu]Lu-PSMA-617 until death.
3. To determine the PFS defined as the date of the first cycle injection of [^177^Lu]Lu-PSMA-617 to the date of first evidence of progression as defined by either radiographic progression, PSA progression, clinical progression, or death from any cause, whichever occurs first.
  a. Radiographic progression is defined as the date of radiographic disease progression on SPECT/CT by visual increase in tumor volume of 30% or greater compared with baseline cycle 1 ± new sites of disease or new sites of PSMA-negative disease progression on CT (10,12) or on PSMA PET/CT as outlined in RECIP or by apparition of new PSMA-negative disease measurable on CT (13). Response assessment criteria using SPECT/CT are summarized in Table 1, and response assessment criteria on PET/CT are summarized in Table 2. See sections 6.8 and 6.9 in the study protocol in the supplemental materials.
  b. PSA progression is defined as the date when a 25% or greater increase in PSA and an absolute increase of 2 ng/mL or more from the nadir is documented and confirmed by a second consecutive value obtained 3 or more weeks later. Rises in PSA within the first 12 wk will be ignored in the absence of other evidence of disease progression in accordance with the Prostate Cancer Clinical Trials Working Group recommendations. Where no decline from baseline is documented, PSA progression is defined as a 25% increase from the baseline value along with an increase in absolute value of 2 ng/mL or more after 12 wk of treatment.
  c. Unequivocal clinical progression is defined as follows:
    i. Marked escalation in cancer-related pain that is assessed by the investigator to indicate the need for other cytotoxic systemic therapy for prostate cancer.
    ii. Immediate need for initiation of new anticancer treatment, surgical or radiologic intervention for complications due to tumor progression even in the absence of radiologic progression.
    iii. Marked deterioration in Eastern Cooperative Oncology Group (ECOG) performance status to grade 3 or greater or in the opinion of the investigator ECOG deterioration indicates clinical progression.
    iv. In the opinion of the investigator, it is in the best interest of the patient to discontinue treatment due to clinical progression.
4. To determine disease control rate defined as the proportion of patients achieving radiographic stable disease (SD), partial response (PR), or CR. CR is defined as absence of a PSMA-avid tumor on either imaging modality. On SPECT/CT, PR is defined as a greater than 30% reduction in visual tumor volume at all sites of involved disease compared with baseline cycle 1, no new sites of PSMA-avid tumor, and no new sites of PSMA-negative tumor on CT; and SD is defined as no change greater than 30% in visual tumor volume compared with baseline cycle 1, no new sites of PSMA-avid tumor, and no new sites of PSMA-negative tumor on CT. Respective response groups on PSMA PET/CT are defined by RECIP (13).
5. To determine disease control rate by combined radiographic plus PSA response with response defined as PSA decline of 50% or more or radiographic PR/CR as defined above.
6. To determine changes in of HRQoL using the following:
  a. The Brief Pain Inventory–Short Form. Pain response defined by improvement from baseline (all patients with ≥4/10) of at least 2-point absolute improvement without an overall increase in opiate use.
  b. Changes in performance status (ECOG).
  c. The FACT-RNT questionnaire (14).

**TABLE 1.** SPECT/CT Response Assessment Criteria

SPECT/CT	CR	PR	SD	PD
Response assessment criteria adopted from Emmett et al. (10)	Absence of any PSMA uptake, no new sites of PSMA-negative tumor	>30% decrease in PSMA-VOL, no new sites of PSMA-positive or PSMA-negative tumor	No change >30% in PSMA-VOL, no new sites of PSMA-positive or PSMA-negative tumor	>30% increase in PSMA-VOL ± new sites of PSMA-positive or PSMA-negative tumor, or new sites of PSMA-negative tumor on CT

SPECT/CT Response Assessment Criteria.

**TABLE 2.** PET/CT Response Assessment Criteria

PET/CT	CR	PR	SD	PD
Response assessment criteria adopted from Gafita et al. (RECIP) (13)	Absence of any PSMA uptake, no new sites of PSMA-negative tumor	≥30% decrease in PSMA-VOL without new sites of PSMA-positive or PSMA-negative tumor	<30% decrease in PSMA-VOL ± new sites of PSMA-positive or PSMA-negative tumor *or* <20% increase in PSMA-VOL ± new sites of PSMA-positive or PSMA-negative tumor *or* PR with new lesions *or* PD without new lesions	≥20% increase in PSMA-VOL with new sites of PSMA-positive or PSMA-negative tumor

## ELIGIBILITY CRITERIA

### Inclusion Criteria

1. Patients with mCRPC.
2. Patients must have received at least one regimen of chemotherapy for mCRPC.
3. Patients must have received at least one androgen receptor signaling inhibitor.
4. Patients must be eligible by PSMA PET VISION criteria (2). PSMA PET/CT must be performed within 8 wk of planned first cycle of [^177^Lu]Lu-PSMA-617.
5. Patients must have sufficient bone marrow capacity as defined by a white blood cell count of at least 2,500/μL, platelet count of at least 100,000/μL, hemoglobin of at least 9.0 g/dL, and absolute neutrophil count of at least 1,500/μL.
6. Patients must have an ECOG performance status of 0 to 2.
7. Patients must be adults at least 18 y of age.
8. Patients must have the ability to understand and sign an approved informed consent form and comply with all protocol requirements.

### Exclusion Criteria

1. Prior [^177^Lu]Lu-PSMA-617 therapy.
2. Less than 6 wk between last myelosuppressive therapy (including docetaxel, cabazitaxel, ^89^Sr, ^153^Sm, ^186^Re, ^188^Re, ^223^Ra, hemibody irradiation) and first cycle of [^177^Lu]Lu-PSMA-617 therapy.
3. Estimated glomerular filtration rate of less than 50 mL/min.
4. Urinary tract obstruction or marked hydronephrosis.

## INVESTIGATIONAL PLAN

A schedule of assessments is provided in the full study protocol in the supplemental materials. The study procedures are described in section 6 of the study protocol.

## TREATMENT DRUG

[^177^Lu]Lu-PSMA-617 will be supplied by Novartis. For further details regarding packaging, storage, administration, accountability, and product retention please see section 4.2 of the study protocol in the supplemental materials.

## INVESTIGATIONAL ARM TIMELINE

In the investigational arm (*n* = 45), patients will receive intravenous injection of 7.4 GBq (±10%) of [^177^Lu]Lu-PSMA-617 once every 6 wk (±1 wk) for a maximum of 12 cycles until disease progression (except for progression during treatment holiday periods which will lead to treatment continuation) or unacceptable toxicity.

**FIGURE 2.**
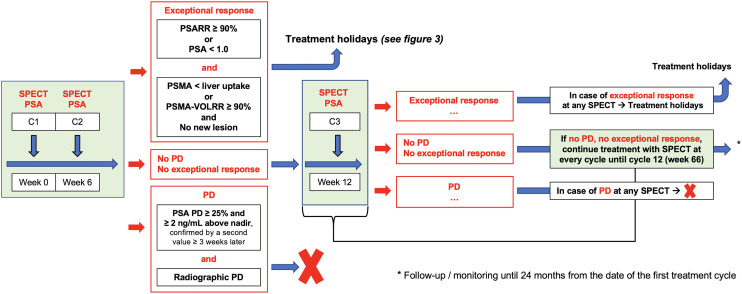
Investigational arm. Patients in investigational arm are treated with up to 12 cycles including potential “treatment holidays” depending on response. Response assessment is based on SPECT/CT and PSA at each cycle during treatment periods. C = treatment cycle; RR = relative response/response rate.

Evaluation of treatment response is performed using posttreatment SPECT/CT and PSA at every cycle. Radiographic response assessment at every cycle uses posttreatment SPECT/CT of cycle 1 as the baseline. If a patient experiences either a PSA decline of 90% or more or a PSA level of less than 1.0 and CR, or a PSA decline of 90% or more or a PSA level of less than 1.0 and PR plus tumor uptake below liver uptake on either SPECT/CT or PET/CT, the subsequent cycle will be postponed until disease progression. The investigational arm is illustrated in Figure 2.

During the treatment holiday period and after the 12th cycle, the disease control and progression will be assessed every 12 wk by PSA and PSMA PET/CT using RECIP (13). According to RECIP, a CR is defined as the absence of any PSMA-ligand uptake on interim PET/CT (RECIP-CR), a PR is defined as a decline of at least 30% in PSMA volume (PSMA-VOL) and no appearance of new lesions, progressive disease (PD) is defined as an increase of at least 20% in PSMA-VOL and appearance of new lesions (RECIP-PD), and SD (RECIP-SD) is defined as any condition but RECIP-PR or RECIP-PD.

**FIGURE 3.**
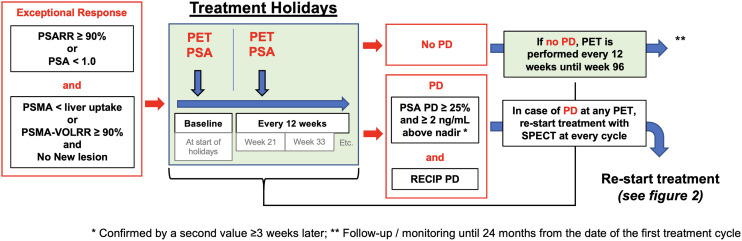
Treatment holidays. Patients with exceptional response in investigational are put on “treatment holidays.” Response assessment during treatment holiday periods is based on PSMA PET/CT and PSA every 12 wk. RECIP = response evaluation criteria in PSMA PET/CT; RR = relative response/response rate; VOL = volume.

The treatment holiday period is illustrated in Figure 3. Once [^177^Lu]Lu-PSMA-617 treatment is restarted after a treatment holiday, evaluation of treatment response is performed using posttreatment SPECT/CT and PSA at each cycle. Radiographic response assessment at each cycle uses posttreatment SPECT/CT of cycle 1 as the baseline.

Safety will be closely monitored following Common Terminology Criteria for Adverse Events with laboratory assessments every 3 wk during the treatment period and during the first 3 mo after the last cycle, and every 3 mo during the remaining follow-up time up to a total of 24 mo. Data will be collected for any adverse events. An interim safety analysis with a stopping rule based on safety outcomes is planned at 50% of enrollment.

Single-time-point SPECT/CT dosimetry protocol (15) at each cycle will be performed to determine the number of cycles that subjects may receive regarding dose-limiting organs. If the calculated kidney biologically effective dose exceeds 39 Gy by dosimetry, the total number of cycles should be adapted and limited to stay under the 39-Gy biologically effective dose limit.

## CONTROL ARM TIMELINE

In the control arm (*n* = 45), patients will receive intravenous injection of 7.4 GBq (±10%) of [^177^Lu]Lu-PSMA-617 once every 6 wk (±1 wk) for a maximum of 6 cycles. Evaluation of treatment response is performed using posttreatment SPECT/CT and PSA every cycle. After termination of [^177^Lu]Lu-PSMA-617 treatment, the disease control and progression will be assessed every 12 wk by PSMA PET/CT and PSA.

## MANAGEMENT OF TOXICITY

Details on toxicity risk reduction and supportive care for [^177^Lu]Lu-PSMA-617 injections, the management of toxicity adverse events, with dosing delays and modification are provided in section 5.3 of the study protocol in the supplemental materials.

## Concomitant Medications

Concomitant first-generation androgen deprivation therapy is allowed. Local external beam radiotherapy, including palliative external radiation, is allowed.

Any other treatments for prostate cancer are not allowed, including switch or initiation of second-generation androgen receptor axis inhibitors (abiraterone, enzalutamide, darolutamide, apalutamide), taxane- or platin-based chemotherapy (washout period of 6 wk before first cycle of [^177^Lu]Lu-PSMA-617 RPT), RPT (including ^89^Sr, ^153^Sm, ^186^Re, ^188^Re, ^223^Ra; washout of 6 wk before first cycle of [^177^Lu]Lu-PSMA-617 RPT), sipuleucel-T, olaparib, rucaparib, and pembrolizumab (washout period of 4 wk before first cycle of [^177^Lu]Lu-PSMA-617 RPT).

Supportive care should be provided as deemed necessary by the treating physician.

## PSMA PET/CT IMAGING

PSMA PET/CT scans as part of the study will be performed with [^68^Ga]Ga-PSMA-11 (injected activity 185 MBq [range, 111–259 MBq], uptake time 50–100 min) with intravenous CT contrast. For more details, see section 6.8 of the study protocol in the supplemental materials.

## ^177^LU-PSMA SPECT/CT IMAGING

Patients will be imaged with posttreatment quantitative SPECT/CT scans at more than 24 h after administration of [^177^Lu]Lu-PSMA-617 (allowed time window for image acquisition, 24–72 h). SPECT/CT scan coverage will extend from midthigh to the vertex with 3 bed positions (∼18 min per bed position, 120 views per bed, 15 s per view). Dosimetry will be performed using MIM software. For more details, see section 6.9 of the study protocol in the supplemental materials.

## STUDY DURATION

Patients may be treated for up to 12 cycles (investigational arm) or 6 cycles (control arm) of [^177^Lu]Lu-PSMA-617 therapy until progressive disease (except for progression during treatment holiday periods in the investigational arm which will lead to treatment continuation), withdrawal of consent, the occurrence of unacceptable toxicity, or a determination by the investigator if the patient is not clinically benefiting.

The duration of subject participation will be from the time of signing informed consent through the 24-mo postinjection visit. The planned duration for the whole study is 48 mo. The study enrollment started in August 2024. Trial completion date is anticipated for December 2028.

## STATISTICS AND SAMPLE SIZE DETERMINATION

The primary endpoint of the study is the 2-y survival rate from the date of the first cycle of [^177^Lu]Lu-PSMA-617 therapy. We assumed that the 2-y survival rate in the control group would be 30%. We hypothesized that the 2-y survival rate in the investigational group would be 55%. A 2-sided log-rank test with an overall sample size of 90 subjects (45 in the control group and 45 in the treatment group) achieves 80.3% power at a 0.05 significance level to detect a hazard ratio of 0.50 when the proportion surviving in the control group is 0.30 and the proportion surviving in the treatment group is 0.55 at 24 mo. The study lasts for 48 mo, of which subject accrual (entry) occurs in the first 12 mo.

The accrual pattern across months is uniform (all periods equal). The proportion dropping out or lost to follow-up in the control group and treatment groups is assumed to be around 6 per group during the study period.

This calculation was performed using power analysis and sample size, and the 2-y survival estimates used in the calculation were estimated from the VISION trial data (2).

Randomization is centrally performed in a 1:1 ratio to the 2 treatment arms. The clinical investigators are blinded to the randomization sequence that has been created upfront in collaboration with the local Department of Medicine Statistics using the software application REDCap (Research Electronic Data Capture) (16). On actual sequential allocation of a patient to a treatment arm, both the study team and the patient will be aware of the treatment arm (open label study).

Efficacy will be reported using descriptive statistics by means of number and percentage of patients dead 24 mo after the first cycle (2-y survival rate). Distribution of safety and tolerability results will be compared between both groups using appropriate tests (e.g., χ^2^ tests). Descriptive analyses (median, standard deviation) will be used to determine OS and PFS; group comparisons will be performed using appropriate tests including survival analyses (e.g., Kaplan–Meier estimator). For more details of the statistical analysis plan, see section 10 of the study protocol in the supplemental materials.

## DISCUSSION

The administration scheme of [^177^Lu]Lu-PSMA-617 used in the VISION trial (amount of injected peptide or ligand [nmol], amount of injected activity [GBq, mCi], time interval between each cycle or fractionation, number of cycles) derives mostly from prior empiric clinical compassionate use of [^177^Lu]Lu-PSMA-617 in Germany (17–19) and prospective trials using other established RPT agents ([^177^Lu]Lu-DOTATATE, ^223^Ra, ^90^Y-ibritumomab-tiuxetan) (20–22). The 6-wk interval selected for this study was based on known hematologic safety considerations (blood count nadir at 3–6 wk after RPT administration) reported in randomized prospective phase 3 trials (20–22). The 7.4-GBq activity regimens were chosen on the basis of dosimetry data (23,24) and the NETTER-1 trial experience (21). The amount of injected activity (GBq, mCi) has been mainly limited by the regulators using external beam radiation therapy dose limits (25). However, these dose limits are potentially overly conservative because of the low dose rate exposure from molecular RPT compared with the high dose rate of external beam radiation therapy. In the posttaxane, postandrogen axis inhibitor setting, the 6 doses of 7.4 GBq of [^177^Lu]Lu-PSMA-617 administered at a 6-wk time interval demonstrated a well established, predictable, well tolerated safety profile. Now that safety is established (2,26), further clinical trials using individualized dosimetry approaches and testing different [^177^Lu]Lu-PSMA-617 therapy administration schemes (activity levels, time intervals) are needed to improve tumor radiation dose delivery and treatment efficacy.

Retreatment (after an interval without ^177^Lu-PSMA, with or without other therapy) and extended regimen (>6 cycles) of ^177^Lu-PSMA therapy have been performed successfully outside of the United States (4–9) with favorable efficacy and toxicity profiles. The concept of treatment holidays in patients experiencing a CR or almost CR before the last cycle 6 (e.g., after cycle 2) has been reported by Emmett et al. and Seifert et al. (7,10). To modify the dosing regimen to the patient’s individual response, early response markers are necessary. A combination of ^177^Lu-PSMA SPECT/CT and early PSA response criteria was used in these retrospective analyses (10,27–31). Patients with marked reduction in PSA/imaging PR who were put on break in treatment until subsequent PSA rise (median of 6.1 mo treatment holidays) had superior survival times compared with patients with stable or reduced PSA or imaging SD who had been treated with the standard regimen of 6 doses of ^177^Lu-PSMA every 6 wk (10). This study substantiates flexible dosing regimens as a promising approach as it achieved similar response rates to the standard regimen while allowing treatment breaks or intensification. We adopted a combination of SPECT/CT and PSA response criteria in the FLEX-MRT study protocol.

## CONCLUSION

The randomized FLEX-MRT trial has the potential to generate evidence for a new individualized treatment strategy using a flexible and extended dosing schedule of ^177^Lu-PSMA RPT in patients with advanced mCRPC.

## DISCLOSURE

FLEX-MRT is an investigator-initiated trial supported by Novartis. Jeremie Calais is the principal investigator. The study protocol has been developed by the investigators without any industrial or other third-party involvement. Adrien Holzgreve is funded by the Deutsche Forschungsgemeinschaft (DFG, German Research Foundation), 545058105, and reports compensation for scientific consulting by ABX advanced biochemical compounds outside of the submitted work. Lena Unterrainer reports funding from Bavarian Cancer Research Center (BZKF) and from the Munich Clinician Scientist Program (MCSP) and reports fees from Novartis (speaker), Telix (consultant), and Astellas Pharma Inc. (speaker) outside of the submitted work. John Nikitas is funded by the Christiaan W. Schiepers Theranostics Fellowship award. Shaojun Zhu reports compensation for regulatory consulting to Freshwind Biotech, SOFIE Bioscience, and AdvanCell isotopes outside of the submitted work. Johannes Czernin is the recipient of a grant from the Prostate Cancer Foundation (2019 Challenge Award, 19CHAL09, and 2017 Challenge Award, 17CHAL02) and the Jonsson Comprehensive Cancer Center NIH–National Cancer Institute Cancer Center Support Grant (P30 CA016042). Johannes Czernin is a founder of SOFIE Biosciences and Infinity TopCo and holds equity in the company and in intellectual property invented by him, patented by the University of California, and licensed to SOFIE Biosciences. He serves as scientific advisor for SOFIE Biosciences. He is a founder of Trethera Therapeutics and holds equity in the company and in intellectual property invented by him, patented by the University of California, and licensed to Trethera. He serves on the scientific advisory board of Aktis Oncology. Jeremie Calais was the recipient of grants from the Prostate Cancer Foundation (2020 Young Investigator Award 20YOUN05), the Society of Nuclear Medicine and Molecular Imaging (2019 Molecular Imaging Research Grant for Junior Academic Faculty), the Philippe Foundation Inc. (New York), and the ARC Foundation (France; International Mobility Award SAE20160604150). Jeremie Calais reports grants from support to his institution from Lantheus, Novartis, and POINT Biopharma. He also reports consulting activities (steering committee, DSMB, advisory boards, speaker, blinded reader) for Advanced Accelerator Applications, Amgen, Astellas, Bayer, Blue Earth Diagnostics Inc., Curium Pharma, DS Pharma, Fibrogen, GE HealthCare, Isoray, IBA RadioPharma, Janssen Pharmaceuticals, Monrol, Lightpoint Medical, Lantheus, Novartis, Pfizer, POINT Biopharma, Progenics, Radiomedix, Sanofi, Siemens-Varian, SOFIE, and Telix Pharmaceuticals, outside of the submitted work. No other potential conflict of interest relevant to this article was reported.
